# ZZ/ZW Sex Determination with Multiple Neo-Sex Chromosomes is Common in Madagascan Chameleons of the Genus *Furcifer* (Reptilia: Chamaeleonidae)

**DOI:** 10.3390/genes10121020

**Published:** 2019-12-06

**Authors:** Michail Rovatsos, Marie Altmanová, Barbora Augstenová, Sofia Mazzoleni, Petr Velenský, Lukáš Kratochvíl

**Affiliations:** 1 Department of Ecology, Faculty of Science, Charles University, 12800 Prague, Czech Republic; marie.altmanova@natur.cuni.cz (M.A.); augstenova.barbora@gmail.com (B.A.); sofia.mazzoleni@natur.cuni.cz (S.M.); kratoch1@natur.cuni.cz (L.K.); 2 Institute of Animal Physiology and Genetics, Czech Academy of Sciences, 27721 Liběchov, Czech Republic; 3 Prague Zoological Garden, 17100 Prague, Czech Republic; velensky@zoopraha.cz

**Keywords:** comparative genome hybridization (CGH), evolution, fluorescence in situ hybridization (FISH), heterochromatin, karyotype, Madagascar, microsatellites, rDNA, sex chromosomes, telomeres

## Abstract

Chameleons are well-known, highly distinctive lizards characterized by unique morphological and physiological traits, but their karyotypes and sex determination system have remained poorly studied. We studied karyotypes in six species of Madagascan chameleons of the genus *Furcifer* by classical (conventional stain, C-banding) and molecular (comparative genomic hybridization, in situ hybridization with rDNA, microsatellite, and telomeric sequences) cytogenetic approaches. In contrast to most sauropsid lineages, the chameleons of the genus *Furcifer* show chromosomal variability even among closely related species, with diploid chromosome numbers varying from 2*n* = 22 to 2*n* = 28. We identified female heterogamety with cytogenetically distinct Z and W sex chromosomes in all studied species. Notably, multiple neo-sex chromosomes in the form Z_1_Z_1_Z_2_Z_2_/Z_1_Z_2_W were uncovered in four species of the genus (*F. bifidus*, *F. verrucosus*, *F. willsii,* and previously studied *F. pardalis*). Phylogenetic distribution and morphology of sex chromosomes suggest that multiple sex chromosomes, which are generally very rare among vertebrates with female heterogamety, possibly evolved several times within the genus *Furcifer*. Although acrodontan lizards (chameleons and dragon lizards) demonstrate otherwise notable variability in sex determination, it seems that female heterogamety with differentiated sex chromosomes remained stable in the chameleons of the genus *Furcifer* for about 30 million years.

## 1. Introduction

Sex determination systems are variable in amniotes; however, this variability is unequally distributed across clades. Several clades such as viviparous mammals, caenophidian snakes, iguanas, softshell turtles, lacertids, and varanids and their relatives exhibited notable stability of sex chromosomes for several dozens of millions of years [1,2,3,4,5,6,7]. As far as is known, the variability in sex determination is concentrated to a few clades such as emydid and geoemydid turtles, geckos, and acrodontan lizards [8,9,10]. The reasons for the differences in the stability of sex determination systems among clades are still not fully understood. Some models suggest that the variability in sex determination is a consequence of the ancestral environmental sex determination (ESD) in a group and multiple independent transitions from ESD to genotypic sex determination (GSD), which is then largely resistant to transitions to ESD [9,10,11,12]. Other scenarios assume that transitions between ESD and GSD are possible in both directions [13,14]. In any case, it seems that highly differentiated sex chromosomes tend to be evolutionary stable and recent molecular evidence disputed earlier reported transitions from GSD with highly differentiated sex chromosomes to ESD [15,16]. On the other hand, poorly differentiated sex chromosomes in reptiles, e.g., in boas and pythons [17,18], tend to be prone to sex chromosome turnovers, but it is not yet clear whether they can be replaced only with other GSD systems or with ESD as well. More accurate testing of these scenarios is currently prevented by the lack of primary data on sex determination in most reptilian lineages.

Iguania is an important squamate group with approximately 1935 species [19]. The two iguanian sublineages, Pleurodonta and Acrodonta, differ in the stability of sex determination. As far as is known, all pleurodontan lizards share male heterogamety, with sex chromosomes being homologous across all iguana families with the exception of basilisks [4,20]. In contrast, species with either ESD, female heterogamety, or male heterogamety were previously reported in Acrodonta (chameleons and agamids). Our knowledge of sex determination in chameleons has, until now, been scarce. Earlier reports on ESD in this group appear questionable [21,22,23]. Male heterogamety with poorly differentiated XX/XY sex chromosomes was found in *Chamaeleo calyptratus* [10], while female heterogamety was reported in *Furcifer oustaleti* and *Furcifer pardalis* [24]. In both species of the genus *Furcifer*, the sex chromosomes are heteromorphic and the W chromosomes are heterochromatic. Notably, *F. pardalis* has multiple neo-sex chromosomes of the type Z_1_Z_1_Z_2_Z_2_/Z_1_Z_2_W [24].

Here, we present the results of our cytogenetic examination based on classical (conventional stain, C-banding) and molecular (comparative genomic hybridization, in situ hybridization with rDNA, microsatellites, and telomeric sequences) cytogenetic approaches in six species of the genus *Furcifer* with the aim of expanding our knowledge of the evolution of karyotypes and sex determination in this group. 

## 2. Materials and Methods 

### 2.1. Samples and Species Verification

Blood samples were collected from six species of chameleons from the genus *Furcifer*, to prepare mitotic chromosome suspensions and DNA isolation (Table 1). Experimental design was performed from accredited researcher (MR: CZ03540, MA: CZ01223, LK: CZ02535), while animals were temporarily kept, when needed, in the animal facility of the Faculty of Science, Charles University (accreditation No. 37428/2019-MZE-18134). All experimental procedures were carried out under the supervision and with the approval of the Ethics Committee of the Faculty of Science, Charles University, followed by the Committee for Animal Welfare of the Ministry of Agriculture of the Czech Republic (permissions No. 35484/2015-16 and 8604/2019-7). All chameleons are kept in Zoo Plzeň, Prague Zoo, and Zoopark Zájezd (Czech Republic).

### 2.2. DNA Isolation and Species Identification

Total DNA was isolated from all examined individuals using the DNeasy Blood and Tissue Kit (Qiagen, Valencia, CA, USA). The standard DNA barcoding region of the mitochondrial gene cytochrome c oxidase I (*COI*) gene was amplified by PCR with primers optimized for reptiles [25] and following standard protocol [26]. The PCR products were purified and sequenced bi-directionally by Macrogen (Seoul, Korea). The obtained *COI* sequences were trimmed in FinchTV [27], aligned in BioEdit v5.0.9 [28], analysed in MEGA v10 [29] and DnaSP v6 [30], and compared to sequences deposited in public databases by BLASTn [31] for accurate taxon identification. All sequences were deposited in GenBank under the accession numbers MN757875–MN757882.

### 2.3. Chromosome Preparation and Staining

The mitotic chromosomal suspensions were prepared by leucocyte cultivation from fresh peripheral blood. Our protocol was previously described in Mazzoleni et al. [32]. Briefly, the cultivation medium (100 mL) was prepared by using 90 mL of commercially prepared D-MEM medium (Sigma-Aldrich, St. Louis, MO, USA), enriched with 10 mL of fetal bovine serum (Gibco, Carlsbad, CA, USA), 3 mL of phytohemagglutinin M (Gibco, Carlsbad, CA, USA), 1 mL of penicillin/streptomycin solution (10000 units/mL; Gibco, Carlsbad, CA, USA), 1 mL L-glutamine solution (200 mM; Sigma-Aldrich, St. Louis, MO, USA), and 1 mL lipopolysaccharide solution (10 mg/mL; Sigma-Aldrich, St. Louis, MO, USA). Then, 100–200 μL of blood were cultivated in 5 mL of the medium for one week at 30 °C. Afterwards, 35 μL colcemid (Roche, Basel, Switzerland) was added 3.5 h before harvesting. The cells were hypotonized in pre-warmed 0.075 M KCl solution for 30 min at 30 °C. Subsequently, the cells were washed four times and stored in Carnoy’s fixative solution (methanol: acetic acid, 3:1).

Chromosomal preparations were dropped to slides and stained by Giemsa for karyotype reconstruction and C-banding for visualization of constitutive heterochromatin, following the protocol of Sumner [33] modified according to Mazzoleni et al. [32].

### 2.4. Fluorescence In Situ Hybridization (FISH) with Telomeric Probe

We applied in situ hybridization with telomeric probe with the aim of uncovering the distribution of telomeric-like sequence (TTAGGG)_n_ in the genomes of the chameleons of the genus *Furcifer*. The probe was prepared and labelled by PCR according to the protocols of Ijdo et al. [34] and Rovatsos et al. [35]. The probe was diluted in hybridization buffer (50% formamide in 2 × saline-sodium citrate (SSC), pH 7.0).

Slides with chromosomal spreads were washed in 2 × SSC solution and subsequently treated with RNase A (100 μg/mL) for 1 h at 37 °C. The slides were again washed in 2 × SSC and treated by 0.01% pepsin for 10 min at 37 °C. Subsequently, the slides were washed in phosphate buffered saline (PBS) solution, post-fixed in 1% formaldehyde solution for 10 min, washed again in PBS, dehydrated in ethanol series and air-dried. The slides were treated in 70% formamide for 4 min at 75 °C, washed in 2 × SSC and again dehydrated in ethanol series. Once the slides were dried, we added the telomeric probe and incubated them overnight at 37 °C. The second day started with washing the slides in 2 × SSC and then three times in 50% formamide for 5 min at 37 °C. In the next step, the slides were washed twice in 2 × SSC and once in 4 × SSC/0.05% Tween 20 (Sigma-Aldrich, St. Louis, MO, USA) solution. After washing, we incubated the slides in 4 × SSC/5% blocking reagent (Roche, Basel, Switzerland) for 45 min at 37 °C. The slides were washed in 4 × SSC/0.05% Tween 20 (Sigma-Aldrich, St. Louis, MO, USA) and incubated for 30 min with avidin-FITC (Vector Laboratories, Burlingame, CA, USA) at 37 °C. The fluorescence signal was twice amplified by the avidin-FITC/biotinylated anti-avidin system (Vector Laboratories, Burlingame, CA, USA). At the end the slides were washed twice in 4 × SSC/0.05% Tween 20 (Sigma-Aldrich, St. Louis, MO, USA), once in PBS and dehydrated by ethanol series. Dried slides were stained by using Fluoroshield with DAPI (4′, 6-diamidino-2-phenylindole) (Sigma-Aldrich, St. Louis, MO, USA).

### 2.5. Fluorescence In Situ Hybridization with rDNA Probe

The probe for FISH with rDNA loci was prepared from a plasmid (pDmr.a 51#1) with an 11.5-kb insert encoding the 18S and 28S ribosomal units of *Drosophila melanogaster* [36] and labelled by dUTP-biotin using nick translation (Abbott Laboratories, Lake Bluff, IL, USA). The rDNA probe was hybridized to chromosome suspensions and detected according to the protocol for FISH with telomeric probes, described above.

### 2.6. Fluorescence In Situ Hybridization with Probes for Repetitive Elements

We tested the distribution of repetitive elements in sex chromosomes of three microsatellite motifs: GATA, AG, and TAC in the species where we had female individuals. Those microsatellite motifs often accumulate in the heterochromatic regions of sex chromosomes in reptiles, including caenophidian snakes [37], chameleons [24], geckos [38], and turtles [39]. For the FISH experiment, we used commercially-synthesized and biotin-labelled probes with sequences (GATA)_8_, (AG)_15,_ and (TAC)_8_ from Macrogen (Seoul, Korea). The probes were diluted in hybridization buffer (50% formamide in 2 × SSC; 10% dextran sulfate; 10% sodium dodecyl sulfate; and Denhard’s buffer, pH 7.0). Slide treatment, hybridization conditions, and signal detection followed the protocol of FISH with telomeric probe with the only difference in post-hybridization washes, where we used 0.4% Nonidet P-40 (Sigma-Aldrich, St. Louis, MO, USA)/2 × SSC and 0.1% Nonidet P-40 (Sigma-Aldrich, St. Louis, MO, USA)/0.4 × SSC solutions instead of 50% formamide/2 × SSC. 

### 2.7. Comparative Genome Hybridization (CGH)

The probes for CGH were prepared by nick translation (Abbott Laboratories, Lake Bluff, IL, USA), according to the manufacturer’s protocol. The probe specific for male genome was labeled by dUTP-biotin (Roche, Basel, Switzerland), while the probe specific for female genome by dUTP-digoxigenin (Roche, Basel, Switzerland). Both probes were mixed in equal concentration and diluted in hybridization buffer (50% formamide in 2 × SSC; 10% dextran sulfate; 10% sodium dodecyl sulfate; and Denhard’s buffer, pH 7.0). The FISH protocol follows the protocol of the FISH with telomeric probe for the slide preparation. The slides were incubated for 48 h at 37 °C. The probe was washed in 50% formamide/2 × SSC three times for 5 min at 37 °C. In the next step, the slides were washed in 2 × SSC at room temperature and 4 × SSC/0.05% Tween 20 (Sigma-Aldrich, St. Louis, MO, USA). After the washes, we incubated the slides in 4 × SSC/5% blocking reagent for 30 min at 37 °C. Subsequently, we incubated the slides with avidin-FITC (Vector Laboratories, Burlingame, CA, USA) and anti-digoxigenin-Rhodamin (Roche, Basel, Switzerland) for 30 min at 37 °C. The slides were washed in 4 × SSC/0.05% Tween 20 (Sigma-Aldrich, St. Louis, MO, USA) and PBS solution and dehydrated in ethanol series. The slides were stained by Fluoroshield with DAPI (Sigma-Aldrich, St. Louis, MO, USA).

### 2.8. Microscopy and Image Analyses

We captured Giemsa-stained metaphases with an Axio Imager Z2 microscope (Zeiss, Oberkochen, Germany), equipped with automatic Metafer-MSearch scanning platform and a CoolCube 1 b/w digital camera (MetaSystems, Altlussheim, Germany). Karyograms were assembled using the software Ikaros (MetaSystems, Altlussheim, Germany). Fluorochrome-stained metaphases were examined under Provis AX70 microscope (Olympus, Tokyo, Japan) equipped with DP30BW camera (Olympus, Tokyo, Japan). The photos from FISH experiments were pseudo-colored and merged in DP Manager (Olympus, Tokyo, Japan). The color of C-banded metaphases was inverted. 

## 3. Results

### 3.1. Furcifer antimena 

**Figure 1:**
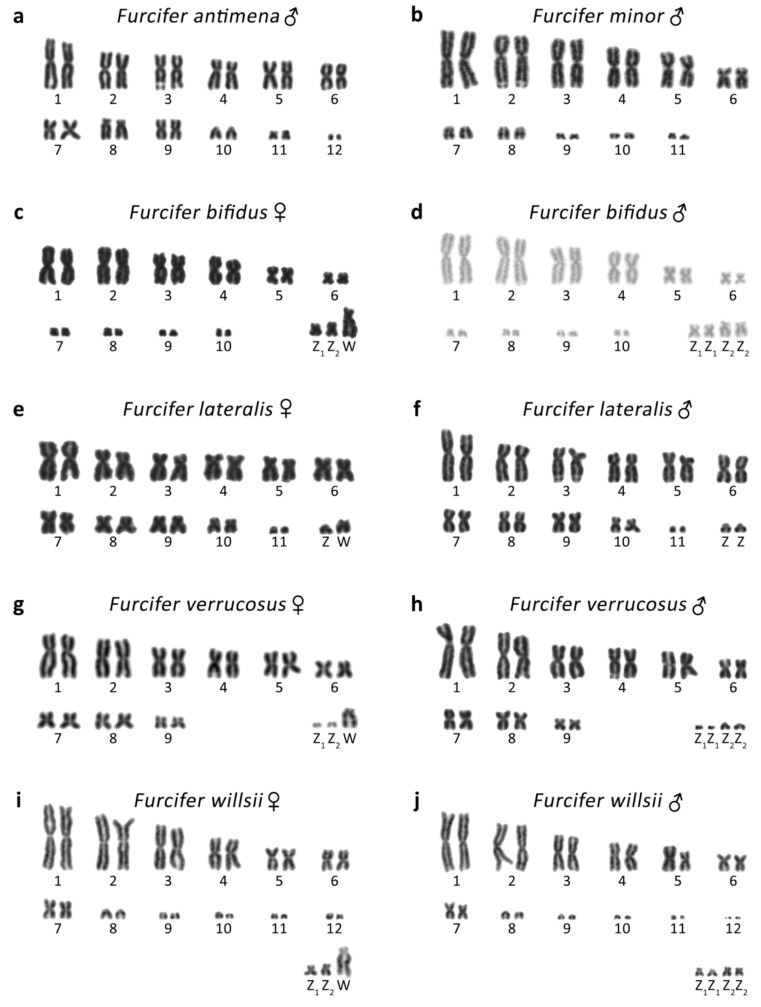
Giemsa-stained karyograms of *Furcifer antimena* (**a**), *F. minor* (**b**), *F. bifidus* (**c**,**d**), *F. lateralis* (**e**,**f**), *F. verrucosus* (**g**,**h**), and *F. willsii* (**i**,**j**). Sex chromosomes are indicated.

**Figure 2:**
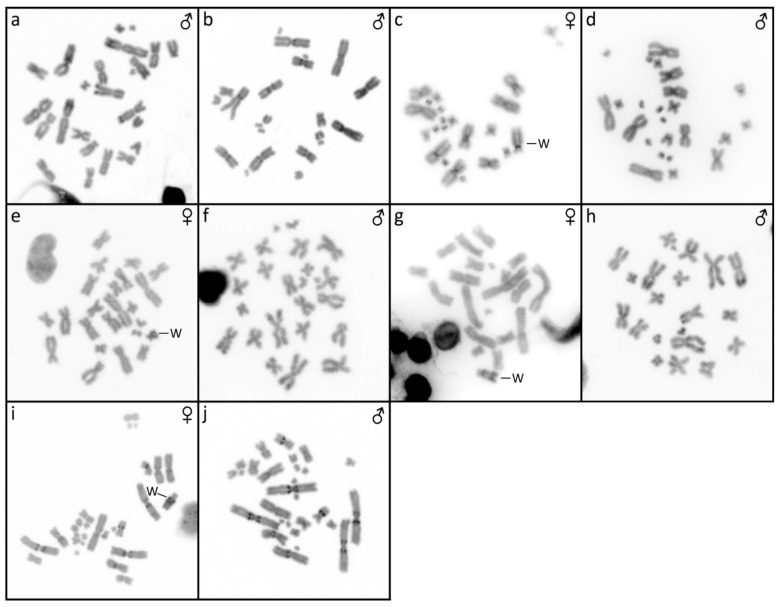
C-banded metaphases of *Furcifer antimena* (**a**), *F. minor* (**b**), *F. bifidus* (**c**,**d**), *F. lateralis* (**e**,**f**), *F. verrucosus* (**g**,**h**), and *F. willsii* (**i**,**j**). W chromosome is marked in females.

**Figure 3:**
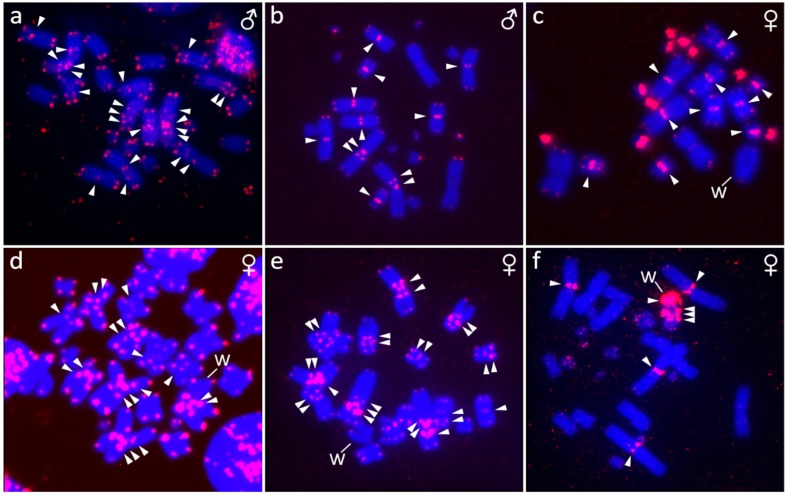
Position of telomeric repeats in metaphases from *Furcifer antimena* (**a**), *F. minor* (**b**), *F. bifidus* (**c**), *F. lateralis* (**d**), *F. verrucosus* (**e**), and *F. willsii* (**f**). Arrowheads point to signals detected by FISH on both arms of a pair of macrochromosomes. W chromosome is marked in females.

**Figure 4:**
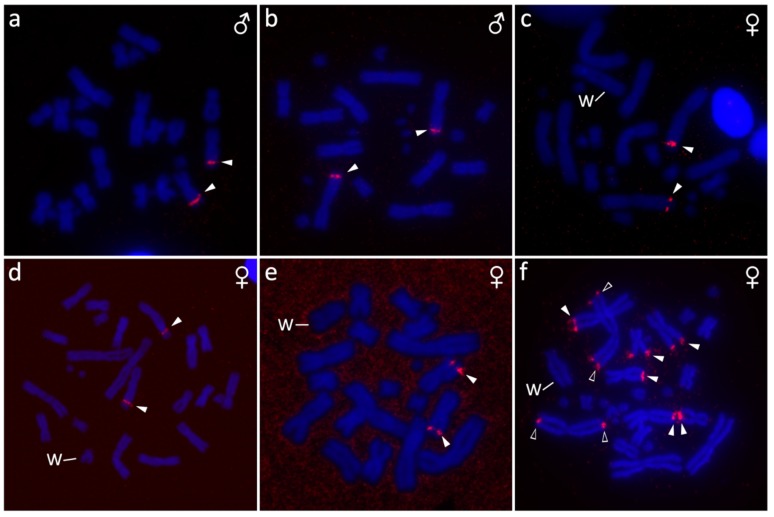
Position of rDNA loci in metaphases of *Furcifer antimena* (**a**), *F. minor* (**b**), *F. bifidus* (**c**), *F. lateralis* (**d**), *F. verrucosus* (**e**) and *F. willsii* (**f**). Arrowheads point to signals detected by fluorescence in situ hybridization (FISH) with empty arrowheads marking signals on both arms of a pair of macrochromosomes. W chromosome is marked in females.

The karyotype consists of 2*n* = 24 chromosomes (Figure 1a), in accordance with the results of Bourgat [40]. C-banding revealed heterochromatin in the centromeric and pericentromeric chromosome regions of many chromosomes (Figure 2a). Only males were examined and no sign of sex chromosomes were identified. The telomeric sequences were detected in terminal positions of all chromosomes and in the centromeric, pericentromeric, and interstitial regions of the sixth largest chromosome pairs (Figure 3a). rDNA loci were located in terminal position of the q arm of the third chromosome pair (Figure 4a).

### 3.2. Furcifer bifidus 

**Figure 5:**
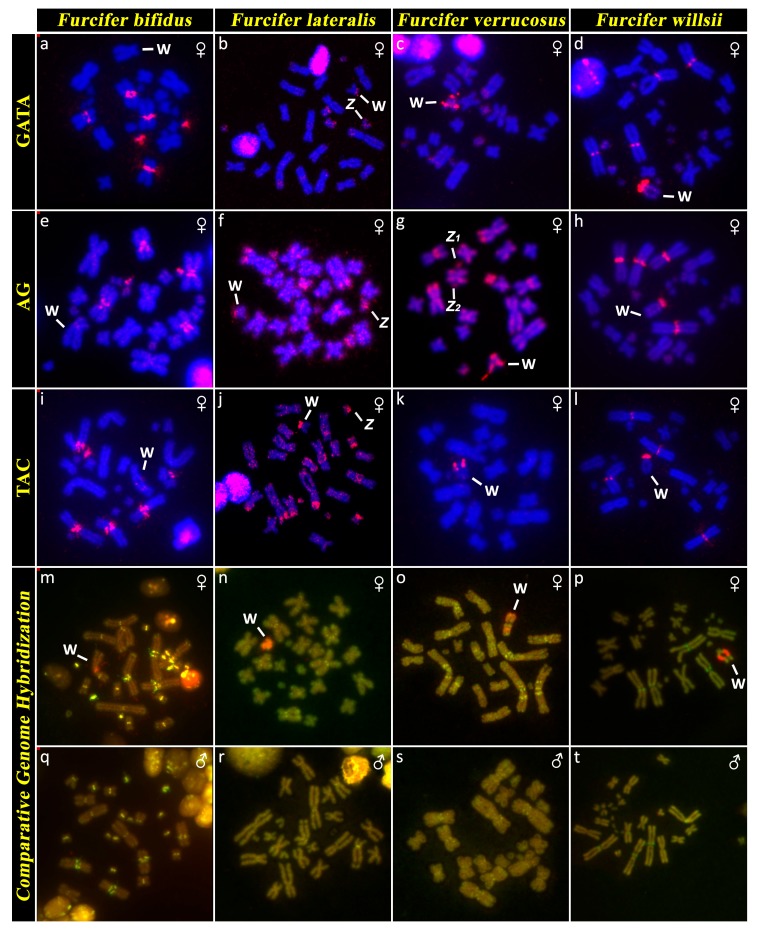
Distribution of the microsatellite motifs GATA (**a**–**d**), AG (**e**–**h**), and TAC (**i**–**l**), and comparative genome hybridization (CGH) (**m**–**t**) in *Furcifer bifidus*, *F. lateralis*, *F. verrucosus,* and *F. willsii*. Sex chromosomes with signal from in situ hybridization are marked. Note that there are no visible sex-specific regions in males (**q**–**t**), in comparison to females, where a female-specific region was detected in the W chromosome (**m**–**p**).

The karyotype consists of 2*n* = 24 chromosomes in males, but 2*n* = 23 chromosomes in females (Figure 1c,d). C-banding revealed distribution of heterochromatin in the centromeric and pericentromeric regions in all autosomes with a notable accumulation on the W chromosome (Figure 2c,d). The sex chromosomes are of Z_1_Z_1_Z_2_Z_2_/Z_1_Z_2_W constitution. Both Z_1_ and Z_2_ chromosomes are microchromosomes, while the W chromosome is medium-sized, subtelocentric, and partially heterochromatic, with a prominent C-positive band in the centromere (Figure 1c,d and Figure 2c,d). The telomeric sequences were detected in the terminal positions of all chromosomes and in the centromeric regions of all chromosomes, except the first pair and the W chromosome (Figure 3c). rDNA loci were located in the terminal position of the q arm of the second chromosome pair (Figure 4c). GATA motif accumulates in three pairs of autosomes, but not in the sex chromosomes (Figure 5a). AG motif accumulates in three pairs of autosomes and in the centromeric region of the W chromosome (Figure 5e). TAC motif accumulates in three pairs of autosomes and in the interstitial regions of the W chromosome (Figure 5i). CGH revealed strong sex-specific differences only in the female in the short arm and/or pericentromeric region of the W chromosome (Figure 5m,q).

### 3.3. Furcifer lateralis

The karyotype consists of 2*n* = 24 chromosomes in both sexes (Figure 1e,f). C-banding revealed distribution of heterochromatin in (peri)centromeric regions in most autosomes and across the whole W chromosome (Figure 2e,f). The sex chromosomes are of the ZZ/ZW type. Both Z and W chromosomes are microchromosomes and the W chromosome is heterochromatic (Figure 2e). The telomeric sequences were detected in the terminal positions of all chromosomes and in the centromeric, pericentromeric and interstitial regions of five chromosome pairs (Figure 3d). rDNA loci were located in the terminal position of the q arm of the third chromosome pair (Figure 4d). GATA motif accumulates in two pairs of microchromosomes and in both sex chromosomes (Figure 5b). AG and TAC motifs accumulate in all autosomes and in both sex chromosomes (Figure 5f,j). CGH revealed strong sex-specific differences only in the female across the whole W chromosome (Figure 5n,r).

### 3.4. Furcifer minor 

The karyotype consists of 2*n* = 22 chromosomes (Figure 1b). C-banding revealed distribution of heterochromatin in the (peri)centromeric chromosome regions (Figure 2b). Sex chromosomes were not identified; however, only males were examined in this species. The telomeric sequences were detected in the terminal positions of all chromosomes, and in the centromeric, pericentromeric, and interstitial regions of five chromosome pairs (Figure 3b). rDNA loci were located in terminal position of the q arm of the second chromosome pair (Figure 4b).

### 3.5. Furcifer verrucosus 

The karyotype consists of 2*n* = 22 chromosomes in males, but 2*n* = 21 chromosomes in females (Figure 1g,h). Karyotype with 2*n* = 22 chromosomes was previously reported for this species from Bourgat [41]. C-banding revealed distribution of heterochromatin in the centromeric and pericentromeric regions in many autosomes and notable block on the W chromosome (Figure 2g,h). The sex chromosomes are of the Z_1_Z_1_Z_2_Z_2_/Z_1_Z_2_W type. Both Z_1_ and Z_2_ chromosomes are microchromosomes, while the W chromosome is small-sized, subtelocentric and partially heterochromatic (Figure 1g and Figure 2g). The telomeric sequences were detected in the terminal positions of all chromosomes and in the centromeric, pericentromeric and interstitial regions of six chromosome pairs (Figure 3e). rDNA loci were located in the terminal position of the q arm of the 4^th^ chromosome pair (Figure 4e). GATA motif accumulates in the W chromosome (Figure 5c). AG motif accumulates in all chromosomes, including the sex chromosomes (Figure 5g). TAC motif accumulates in the W chromosome (Figure 5k). CGH revealed strong sex-specific differences only in females in the short arm and part of the long arm of the W chromosome (Figure 5o,s).

### 3.6. Furcifer willsii 

The karyotype consists of 2*n* = 28 chromosomes in males, but 2*n* = 27 chromosomes in females (Figure 1i,j). A karyotype with 2*n* = 28 chromosomes was previously reported for this species [42]. C-banding revealed distribution of heterochromatin in the (peri)centromeric regions in all autosomes and a large accumulation in the W chromosome (Figure 2i,j). The sex chromosomes are of the Z_1_Z_1_Z_2_Z_2_/Z_1_Z_2_W type. Both Z_1_ and Z_2_ chromosomes are microchromosomes, while the W chromosome is large, subtelocentric, and partially heterochromatic (Figure 1i and Figure 2i). The telomeric sequences were detected in terminal positions of all chromosomes and in the centromeric regions of the first and second chromosome pair and in the W chromosome (Figure 3e). rDNA loci are located in the terminal positions of four chromosome pairs in five clusters (Figure 4f). GATA, AG, and TAC motifs accumulate in the centromeric regions of the first and second chromosome pair and in the p arm of the W chromosome (Figure 5d,h,l). CGH revealed strong sex-specific differences only in the central part of the long arm of the W chromosome (Figure 5p,t).

## 4. Discussion

Chromosome numbers revealed by us in *F. antimena, F. verrucosus,* and *F. willsii* agree with the pioneering descriptions of their male karyotypes performed before the onset of advanced cytogenetic techniques [40,41,42]. According to our knowledge, karyotypes of three species (*F. bifidus, F. lateralis,* and *F. minor*) are described here for the first time. Our study increased the number of the chameleons of the genus *Furcifer* with described karyotypes to 14 (reviewed in [43]) and confirmed that the members of this genus have rather variable karyotypes, with diploid chromosome numbers (2*n*) in males being 2*n* = 22, 24, 26, and 28. Notably, chameleons range in diploid chromosome numbers from 2*n* = 20 to 2*n* = 62 [43] and have generally higher variability in comparison to other closely-related squamate lineages, such as iguanas (2*n* = 19–48) [44,45,46], snakes (2*n* = 24–56) [44,47], and varanids (2*n* = 40 in all studied species) [48]. This variability is visible not only in the chromosomal numbers, but also in differences in size and shape of chromosomes as can be exemplified in the variation of the position of interstitial telomeric repeats (Figure 3) and rDNA loci (Figure 4). Such variation could be the result of multiple inter- and intra-chromosomal rearrangements.

Before our present study, a chromosome system of sex determination was known in only three species of chameleons (*F. oustaleti, F. pardalis,* and *C. calyptratus*) [10,24]. Our data show that female heterogamety is common in the genus *Furcifer*, as we detected cytogenetically distinguishable sex chromosomes in all four species (*F. bifidus*, *F. lateralis*, *F. verrucosus,* and *F. willsii*), with available female specimens. The W chromosomes in all these species contain notable heterochromatic blocks (Figure 2) and CGH revealed that their genetic content is at least partially female-specific (Figure 5) in accordance with the previously published results in *F. oustaleti* and *F. pardalis* [24]. The female-specific signals in CGH co-localize with heterochromatin in the W chromosomes. On the contrary, the XX/XY sex chromosomes in *C. calyptratus* are euchromatic and not detectable by CGH [43,49]. C-banding, CGH, and FISH with telomeric repeats did not detect any clear sex-specific differences in karyotypes of several other species of chameleons from the genera *Calumma, Rieppeleon,* and *Trioceros* [43]. It seems that female heterogamety with a distinct W sex chromosome is an apomorphy of the clade containing the studied members of the genus *Furcifer* [50,51,52,53]. Only limited (AG and TAC) or even lack (GATA) of accumulations of microsatellite motifs were observed on the W chromosome in *F. bifidus*, but notable accumulation of all three motifs was observed in *F. verrucosus*, *F. lateralis*, and *F. willsii*, suggesting that the W chromosomes are homologous in these three species, with the accumulations emerging in their common ancestor after their split from *F. bifidus*. Despite this variability in the W chromosomes, the Z chromosomes seem to have similar morphology in all examined species. Therefore, we assume that female heterogamety was already present in the common ancestor of the studied members of the genus *Furcifer* living approximately 30 million years ago (dating follows [51]).

**Figure 6:**
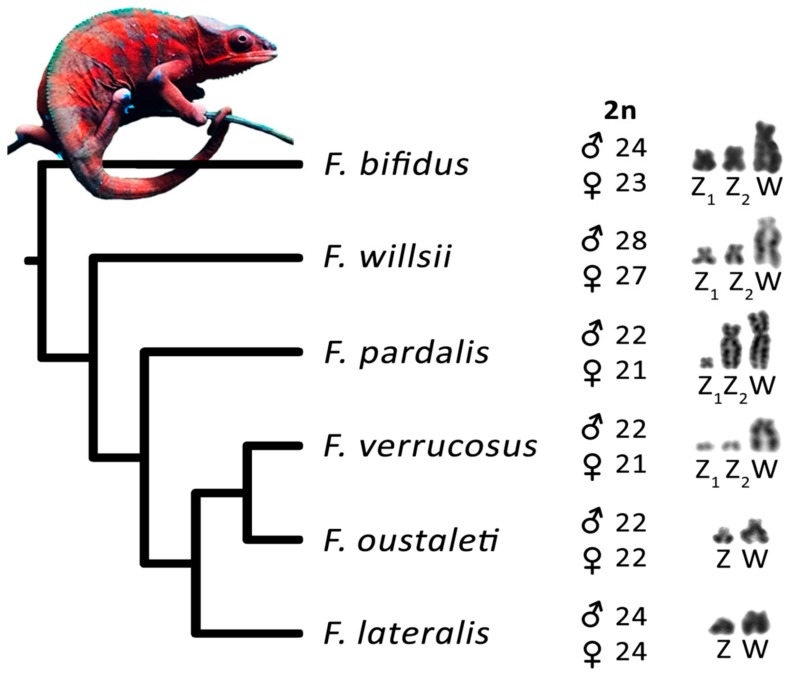
Phylogenetic relationships of the studied species of the genus *Furcifer* following Pyron and Burbrink [51]. Diploid chromosome numbers and sex chromosome constitution are illustrated.

In vertebrates, multiple neo-sex chromosomes evolve frequently under male heterogamety, but rarely under female heterogamety as documented in mammals, birds, reptiles, and fishes [54,55]. Several hypotheses were suggested to explain this pattern, including stronger sexual selection in males reducing effective population size for the Y, male-biased mutation rates [55], and the preferential segregation against neo-sex chromosomes from meiotic drive in female meiosis [54]. The latter hypothesis suggests that rearrangements connected with the emergence of multiple neo-sex chromosomes under female heterogamety could result in a biased sex ratio due to the female meiotic drive, which should be penalized by selection for equal sex ratio. According to this hypothesis, the fixation of the rearrangements of the Y chromosome leading to multiple neo-sex chromosomes can be fixed in a population more frequently, as this male-specific chromosome is not involved in female meiosis and is therefore sheltered against the effects of the female meiotic drive affecting the X, Z, and W chromosomes and autosomes. Interestingly, four species of the genus *Furcifer* (*F. bifidus*, *F. verrucosus*, *F. willsii*, and *F. pardalis*) with female heterogamety possess multiple sex chromosomes. Multiple sex chromosomes can arise either by fusion of the ancestral W chromosome with an autosome or by fission of the ancestral Z chromosome. In the members of the genus *Furcifer* with multiple sex chromosomes, the W is always a large chromosome, therefore, we assume that their multiple sex chromosomes were formed by fusions. Even more interestingly, four species with multiple neo-sex chromosomes do not form a monophyletic clade in respect to the two species with simple ZZ/ZW sex chromosomes (*F. lateralis* and *F. oustaleti*) in the reconstructed phylogeny of the genus (Figure 6). We assume that the Z_1_Z_1_Z_2_Z_2_/Z_1_Z_2_W neo-sex chromosomes were formed independently several, possibly even four times, from the ancestral simple ZZ/ZW system. However, we cannot exclude a scenario that simple ZZ/ZW sex chromosome systems are derived and emerged within the genus via chromosomal fissions from the ancestral multiple neo-sex chromosomes. These alternatives should be investigated in future by identification of the gene content of the sex/neo-sex chromosomes, allowing the assessment of their homology. The genus *Furcifer* together with elapid sea snakes, the other reptilian lineage with multiple emergences of neo-sex chromosomes under female heterogamety [54], can serve as excellent models to investigate evolutionary hypotheses on the origin of multiple neo-sex chromosomes. 

## Figures and Tables

**Table 1:** Number of individuals per species and sex, analyzed in this study.

Species	Sex	
	♂	♀
*Furcifer antimena*	4	0
*Furcifer bifidus*	2	2
*Furcifer lateralis*	2	2
*Furcifer minor*	3	0
*Furcifer verrucosus*	3	4
*Furcifer willsii*	2	2
